# Supplementary material for: Neighborhood nonnegative matrix factorization identifies patterns and spatially-variable genes in large-scale spatial transcriptomics data
Source: Genome Biol. 2026 Jan 16;27:31. doi: 10.1186/s13059-025-03846-6 (PMC12892798; doi:10.1186/s13059-025-03846-6)
Supplement: Supplementary file 1 — Additional file 1. Contains Supplemental Figures S1-S4, including AIC graphs for all three datasets and NNMF signature activity for signatures 1-30 on the CRC MERFISH data. [file 13059_2025_3846_MOESM1_ESM.pdf]

Additional file 1 for *Neighborhood nonnegative matrix factorization identifies patterns and spatially-variable genes in large-scale spatial transcriptomics data*

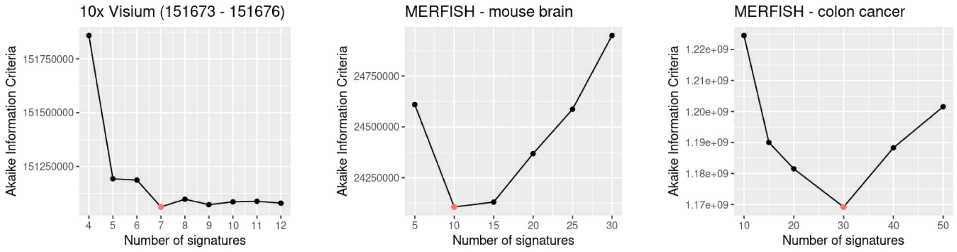

Figure S1: Recovering the optimal number of signatures for the three datasets using Akaike information criterion (AIC), where the optimal number (lower AIC is better) is marked with a red dot.

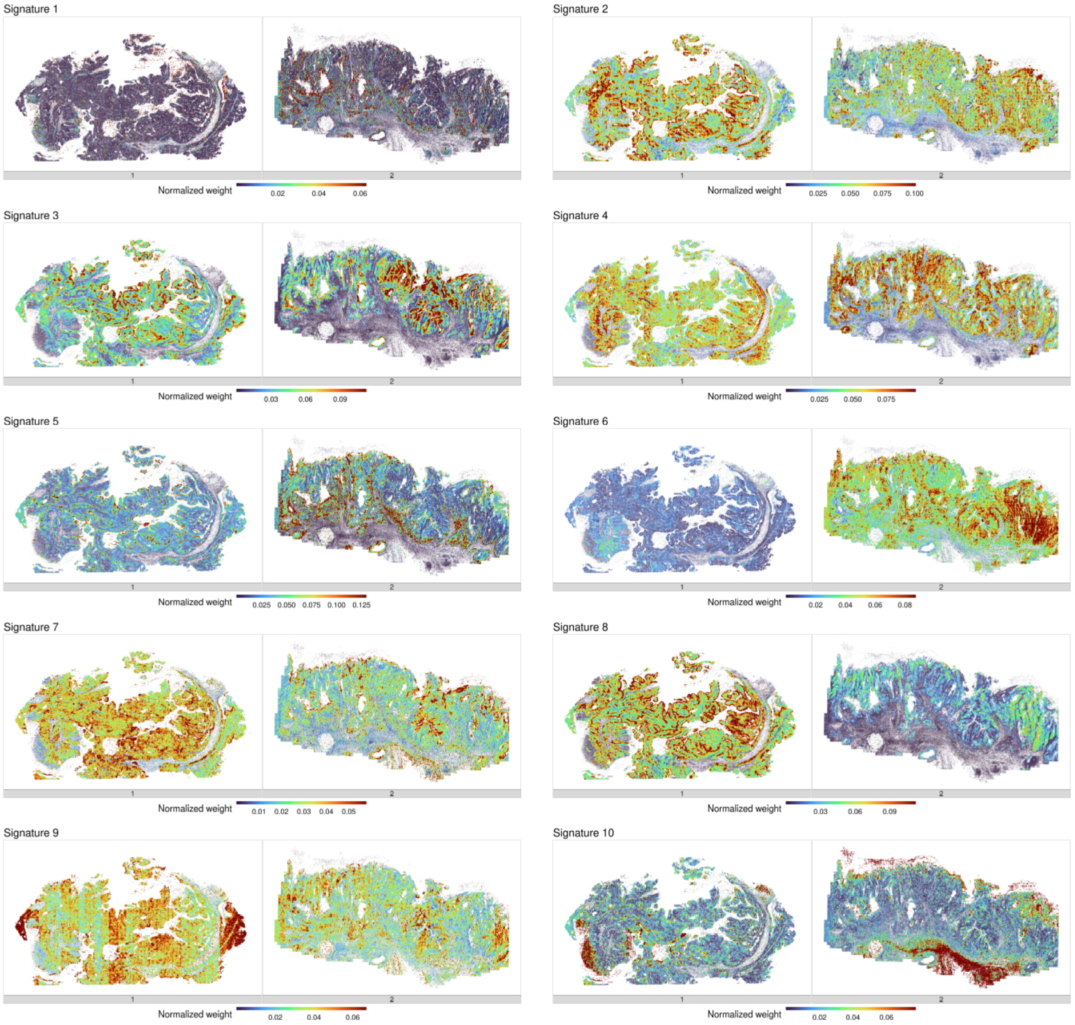

Figure S2: **NNMF signature activity.** Top signatures for the CRC Merfish data: signatures 1-10.

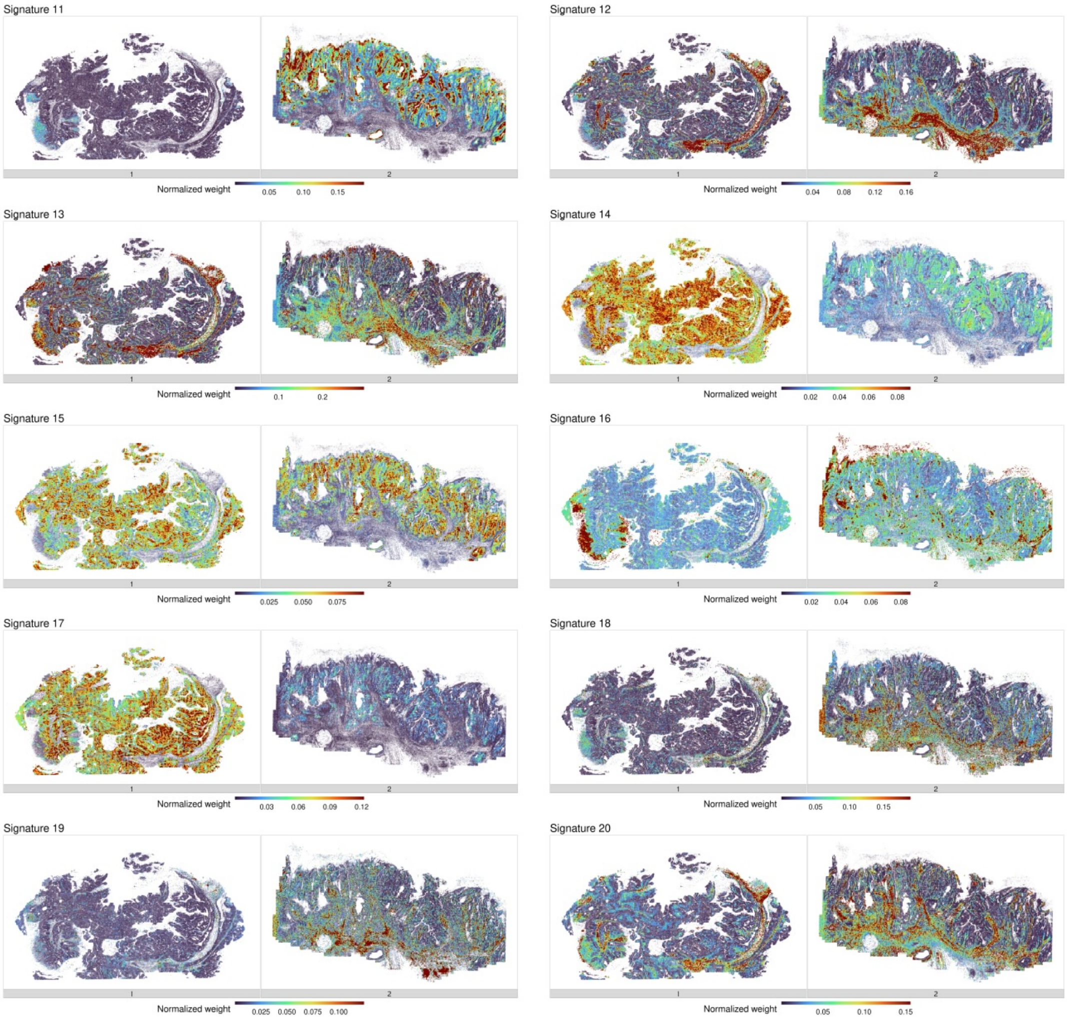

Figure S3: **NNMF signature activity.** Top signatures for the CRC Merfish data: signatures 11-20.

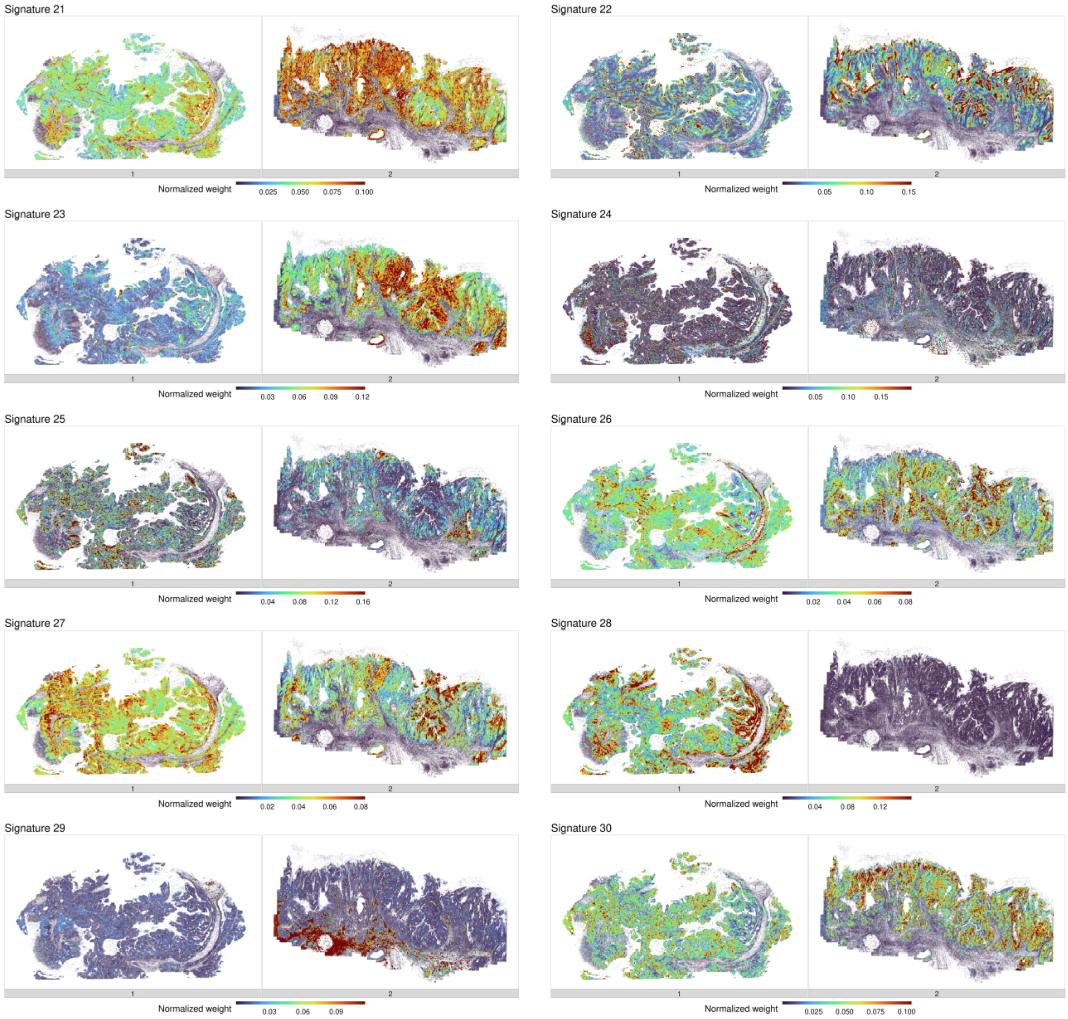

Figure S4: **NNMF signature activity.** Top signatures for the CRC Merfish data: signatures 21-30.
